# Supplementary material for: Role of Doping Agent Degree of Sulfonation and Casting Solvent on the Electrical Conductivity and Morphology of PEDOT:SPAES Thin Films
Source: Polymers (Basel). 2021 Feb 23;13(4):658. doi: 10.3390/polym13040658 (PMC7926741; doi:10.3390/polym13040658)
Supplement: Supplementary file 1 [file polymers-13-00658-s001.pdf]

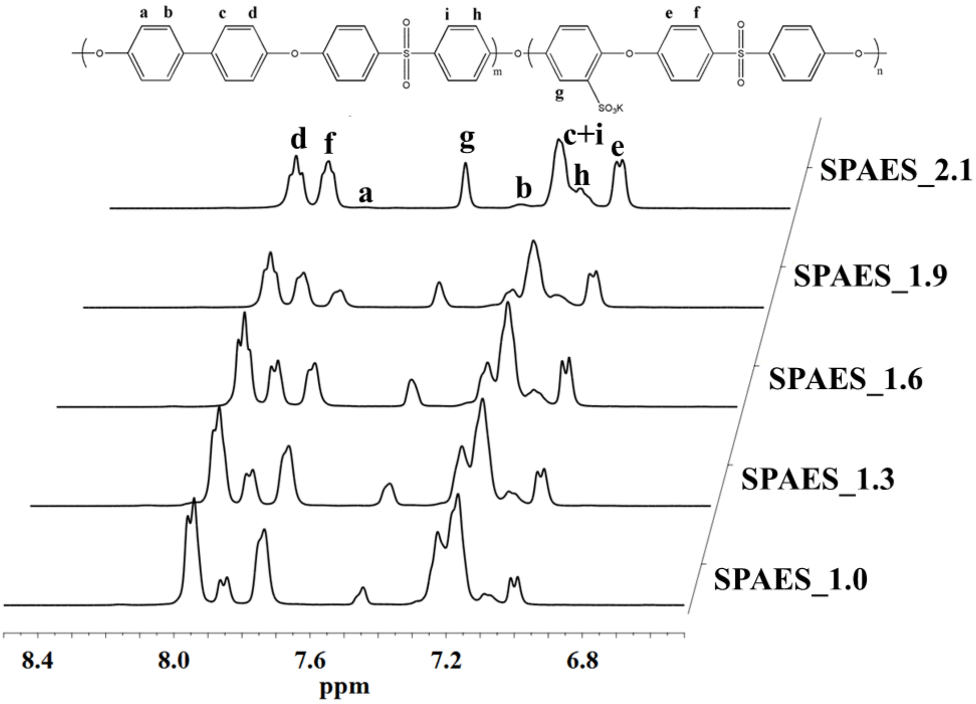

Figure S1. ^{1}H-NMR spectra aromatic region of linear SPAES_1.0 and branched SPAES_1.3, 1.6, 1.9 and 2.1.

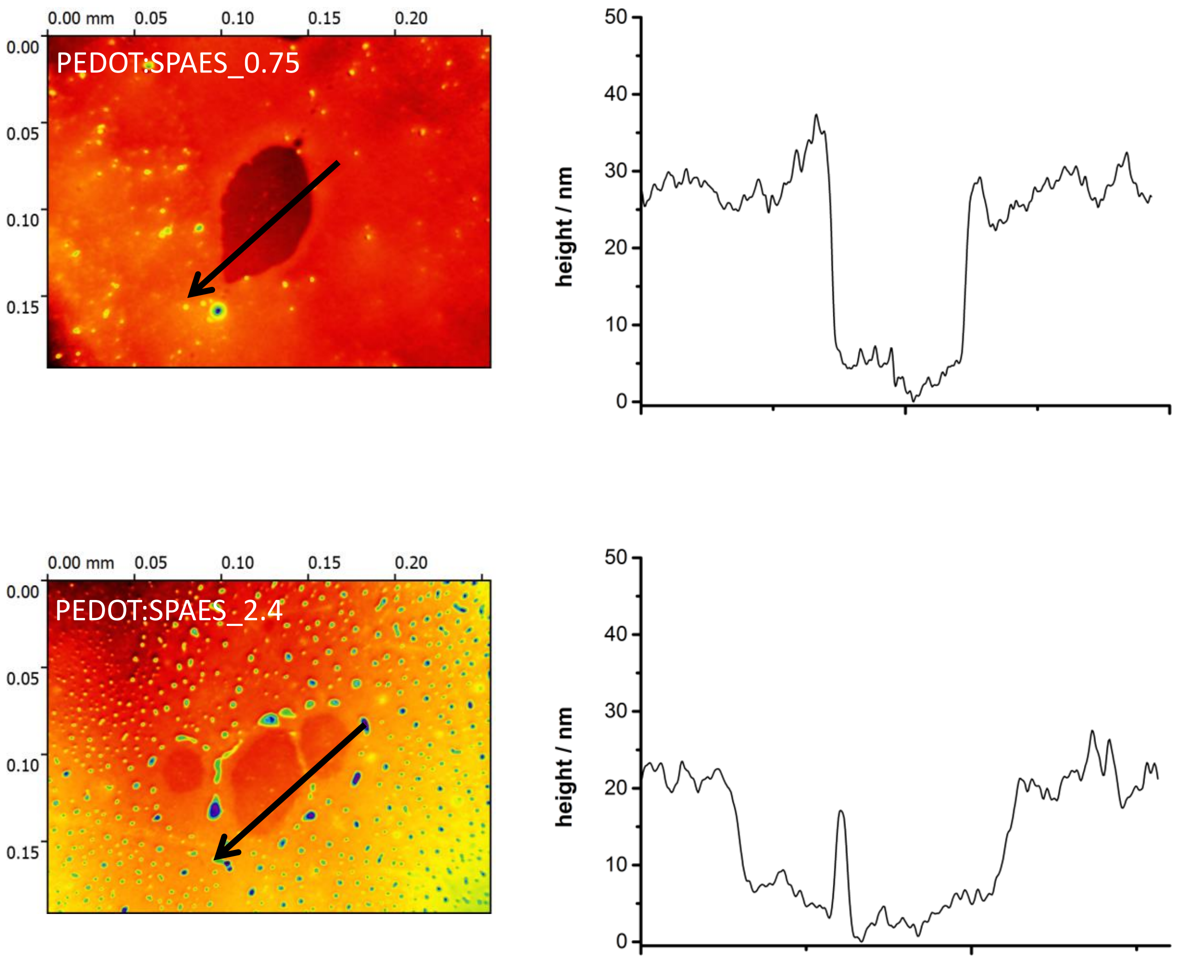

Figure S2. Confocal laser scanning microscope images and height profile of PEDOT:SPAES in DMSO films with a) DS = 0.75 and b) DS= 2.4 (meq R-SO_3^-×g^{-1} of polymer).
